# Enhancing Diagnostic Quality of Intra-Oral Radiographs Through Targeted Training Interventions and Super User Integration

**DOI:** 10.7759/cureus.80076

**Published:** 2025-03-05

**Authors:** Saffa Dean, Ravi Rathod, Hitesh Bansal

**Affiliations:** 1 Oral Surgery, Barts Health NHS Trust, London, GBR; 2 Oral and Maxillofacial Surgery, Barts Health NHS Trust, London, GBR

**Keywords:** dental x-ray, diagnostic, intra-oral radiography, super user, training intervention

## Abstract

Dental and oral and maxillofacial surgery professions rely on intra-oral radiographs to effectively diagnose and carry out necessary treatment.

A retrospective comparative analysis was carried out on a sample of digital radiographs from two sites within the same National Health Service (NHS) trust. Images were graded as per Faculty of General Dental Practitioners (FGDP) guidelines as either ‘Acceptable’ or ‘Not Acceptable’. The FGDP state that 95% of digital radiographs taken within a department should be rated as "acceptable” in terms of diagnostic quality. Diagnostic quality was assessed based on the ability to identify structures necessary for treatment and whether the radiographs had to be retaken. Additionally, surveys were given out to both the medical and dental radiographers across both sites to gather data on their level of training in dental radiography, experience and confidence.

The results revealed significant disparities in diagnostic quality of radiographs across both sites and training events and confidence levels between medical and dental radiographers.

This paper outlines strategic improvements to the radiography team training, the establishment of a super user system to standardize training quality, and the introduction of a shared rota to enhance clinical efficiency.

These initiatives were placed in order to improve radiograph quality and boost the confidence of medical radiographers in intra-oral imaging, ultimately improving patient care and outcomes and reducing their exposure to excessive radiation.

## Introduction

The electromagnetic spectrum contains a range of different types of radiation from radio waves to gamma rays. X-rays are a type of ionising electromagnetic radiation and therefore have the potential to cause harm [1].

The average person in the United Kingdom (UK) is exposed to approximately 2700 µSv per year through background radiation alone [2]. The effective dose from an intraoral periapical radiograph (IOPA) is approximately 2 µSv if using digital films and a rectangular collimator [3]. Using a rectangular collimator effectively is more operator-dependent, however, there are findings that suggest that reduces radiation dose significantly [4]. Despite the dose being fairly low risk, repetitions of radiographs due to non-diagnostic quality will still increase the risks of radiation to patients [5-6].

According to the Royal College of Radiologists (RCR), “a useful investigation is one in which the results, positive or negative, will aid clinical management and/or add confidence to the diagnosis” [7]. Intraoral periapical (IOPA) and orthopantomogram (OPG) radiographs are essential tools in dental and oral and maxillofacial surgery (OMFS) professions for diagnosis, treatment planning and review [8]. IOPAs and OPGs can be utilised for a multitude of reasons including assessing bone height, periapical pathology, evaluating root morphology and also post-operative assessment of implant placement and healing of lesions [9].

The Faculty of General Dental Practice (FGDP) states that 95% of digital radiographs taken within a department should be rated as "acceptable” in terms of diagnostic quality [10]. Using these guidelines a recent analysis was carried out across two hospitals within the same NHS trust - Whipps Cross Hospital (WXH), a district general hospital, with medical radiographers, and Royal London Hospital (RLH), a teaching hospital with dental radiographers.

This paper aims to address these differences through targeted improvements in radiography training, the implementation of a super user system, and a shared rota between the radiography and OMFS teams at WXH.

## Materials and methods

A retrospective analysis of 100 digital radiographs taken between January 2024 and June 2024 across both WXH and RLH was carried out. This included 25 OPGs and 25 IOPAs from each of the two hospital sites. These radiographs were evaluated alongside a review of the patient notes to provide a comprehensive analysis. The radiographs were assessed based on the clinical question stated in the imaging request and graded as either “acceptable” or “not acceptable” as per the FGDP guidelines [8]. The grading process focused on identifying necessary anatomical structures required for diagnosis and treatment, such as the full visibility of the correct tooth and adequate periapical regions beyond root apices [11].

The specific justification requests for each radiograph were analysed to gain a better understanding of what needed to be seen in the image to confirm its diagnostic status. The majority of the intra-oral radiographs were requested for assessing periapical pathology, planning extractions, and examining impacted teeth. The OPGs were requested for similar reasons as well as broader assessments, including the evaluation of temporomandibular joints, assessment of lower third molars and proximities of these teeth to the inferior dental nerve [12].

We also took note of any radiographs that required repetition in order for clinicians to diagnose effectively.

A questionnaire was distributed to the radiographers across both sites to evaluate their training history, clinical exposure, and confidence in performing intra-oral radiography and taking OPGs. The survey was identical for both sites, ensuring uniformity in the data collected. It comprised questions covering the frequency of taking radiographs, last training session attended, confidence levels in taking diagnostic-quality radiographs and the perceived need for further training.

A total of 17 responses were collected from medical radiographers at WXH, compared to seven responses from dental radiographers at RLH. The survey results were analysed to identify gaps in training and confidence, which allowed the development of targeted interventions between the OMFS and radiography teams.

Several interventions were introduced to address the disparities observed in radiograph qualities and radiographer confidence. A tailored training day was established at WXH. This involved theoretical lecture-based teaching followed by a hands-on practical session, using the same equipment available in the department that is used on patients during clinics. The main teaching focus was to demonstrate the placement of intra-oral radiograph films and positioning of the X-ray beam. Training was carried out on volunteers taking part in the session. Positioning was confirmed by the training leads and no participants were radiated during the training. This approach aimed to familiarise radiographers with the equipment and optimise their clinical skills.

“Super Users” are specifically trained users within a department. These are valuable members of the team who are given dedicated roles and are then able to train other members of the team in that field. “Super Users” have been used in various aspects of health care such as when introducing electronic patient records to an organisation and implementing artificial intelligence into existing frameworks [13-14]. A “super user” system was suggested where experienced radiographers were designated to take dental radiographs. The super users were decided by the WXH radiography team based on their exposure and confidence in taking intra-oral radiographs. These super users are responsible for sharing their knowledge and skills to colleagues, ensuring standardised practices within the department. The super users were also tasked with providing support during busy OMFS clinics, reducing the need for OMFS clinician intervention. To facilitate smooth workflow and ensure ‘super user’ availability during critical times, the OMFS team shared their rota with the radiography team. This collaboration allowed super users to be scheduled during OMFS clinics, minimizing disruptions and enhancing efficiency.

These methods provided a robust framework for identifying, addressing, and monitoring the factors influencing radiograph quality and radiographer confidence, ultimately contributing to improved patient care.

## Results

The results revealed significant differences in the quality of intra-oral radiographs across the two sites, with WXH performing below the FGDP standard.

At RLH, 24 (96%) of the intra-oral radiographs were rated as "acceptable," meeting the FGDP guidelines. In contrast, only 19 (76%) of the intra-oral radiographs taken at WXH were of acceptable diagnostic quality. Our findings also highlighted that far fewer IOPAs were taken at WXH in comparison to RLH, which may provide an insight into these findings.

All OPGs taken across both sites were deemed diagnostically acceptable.

Of the 6 (34%) IOPAs at WXH that were not diagnostic, all of them were retaken to allow the clinical question to be answered, which increases the radiation risk to the patient due to increased radiation exposure and the potential complications this can cause.

Through our investigation using clinical notes, we realised that there were often times, clinicians within the OMFS team would be required to abandon busy clinics in order to take IOPAs within the radiography department.

Survey data highlighted that WXH radiographers were less experienced and confident in taking intra-oral radiographs compared to their RLH counterparts.

**Figure 1:**
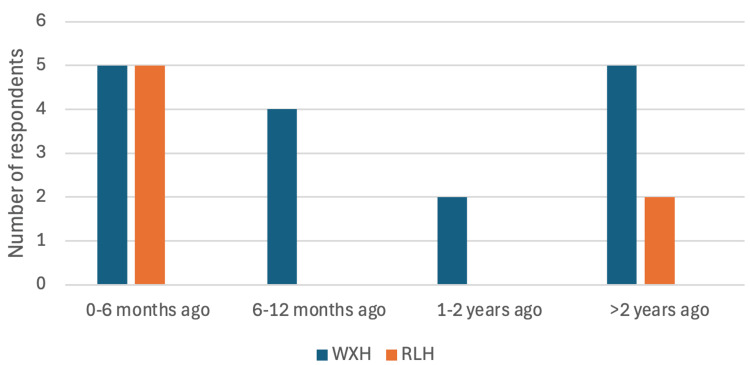
Survey Question: "When was your most recent dental radiography training session?" – Bar chart comparing responses between WXH and RLH radiographers. WXH: Whipps Cross Hospital; RLH: Royal London Hospital

Specifically, 11 (65%) of the WXH radiographers had not received training in dental radiography for over six months compared to 5 out of the 7 (71%) of the RLH radiographers who had training within the last six months (Figure 1).

**Figure 2:**
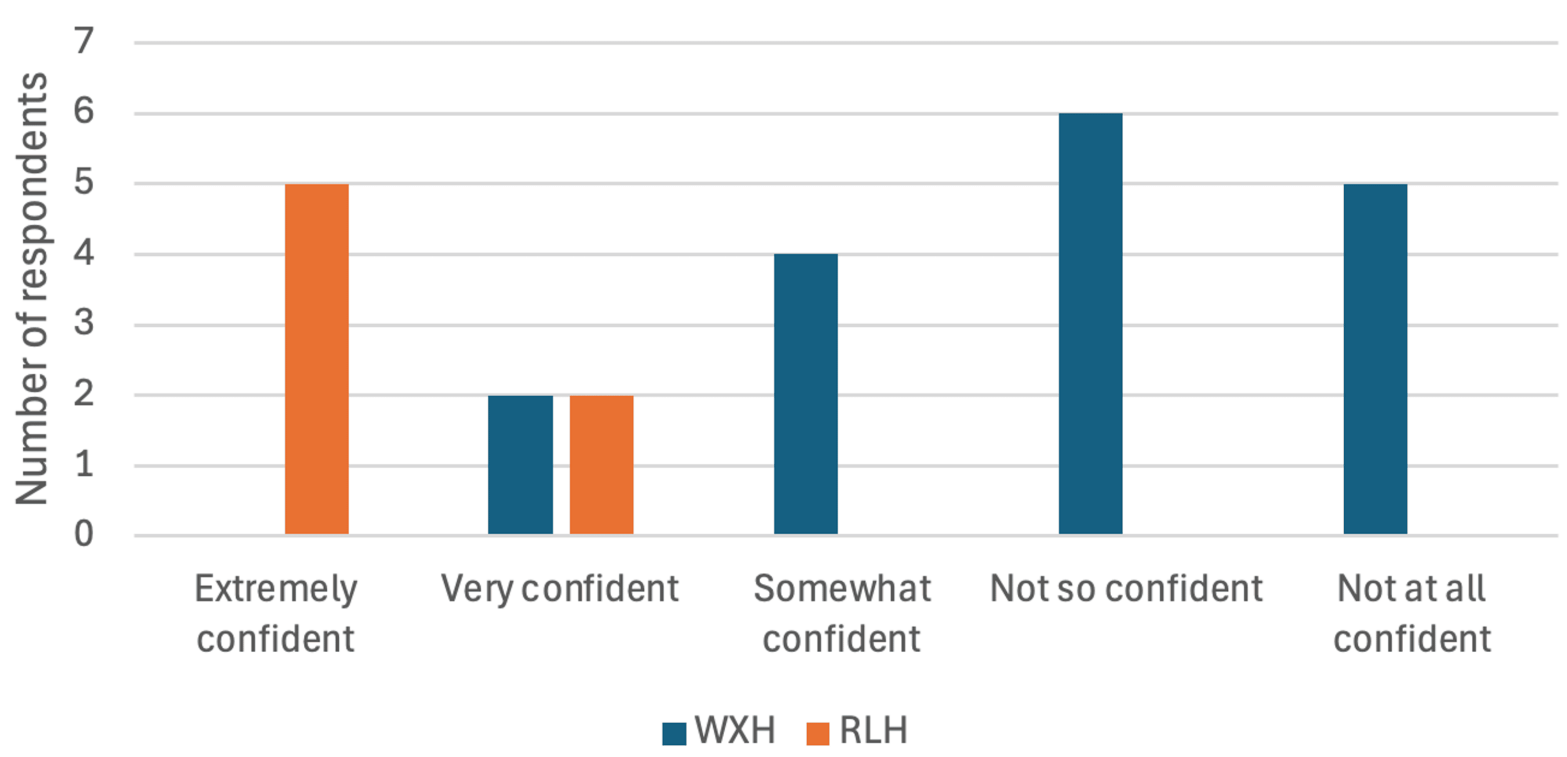
Survey Question: "How confident do you feel taking intra-oral radiographs?" – Bar chart comparing responses between WXH and RLH radiographers. WXH: Whipps Cross Hospital; RLH: Royal London Hospital

Over 50% (11) of those at WXH felt ‘not so confident’ or ‘not confident at all’ when taking IOPAs, whereas all seven of those at RLH felt ‘very confident’ or ‘extremely confident’ when taking intra-oral X-rays (Figure 2).

**Figure 3:**
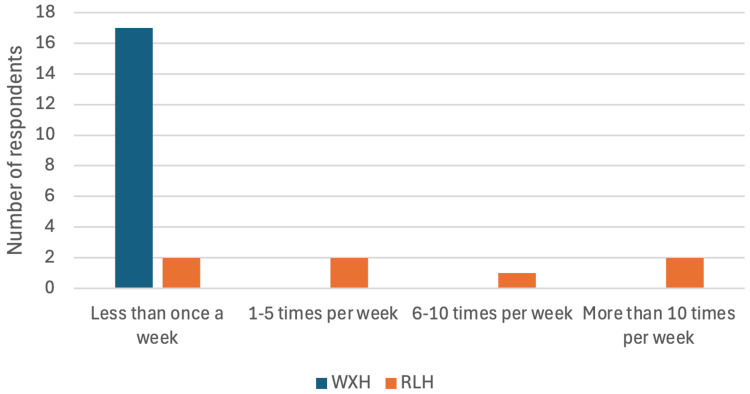
Survey Question: "How often do you take intra-oral radiographs?" – Bar chart comparing responses between WXH and RLH radiographers. WXH: Whipps Cross Hospital; RLH: Royal London Hospital

At WXH, all respondents took intra-oral radiographs less than once per week, whilst 5 (71%) of the RLH team took at least one IOPA per week (Figure 3).

In comparison, 14 (82%) WXH radiographers felt at least ‘somewhat confident’ in taking OPGs and 16 (94%) took them at least once a week. All of the RLH respondents felt ‘extremely confident’ taking OPGs and 6 (85%) of them took OPGs at least once a week.

**Figure 4:**
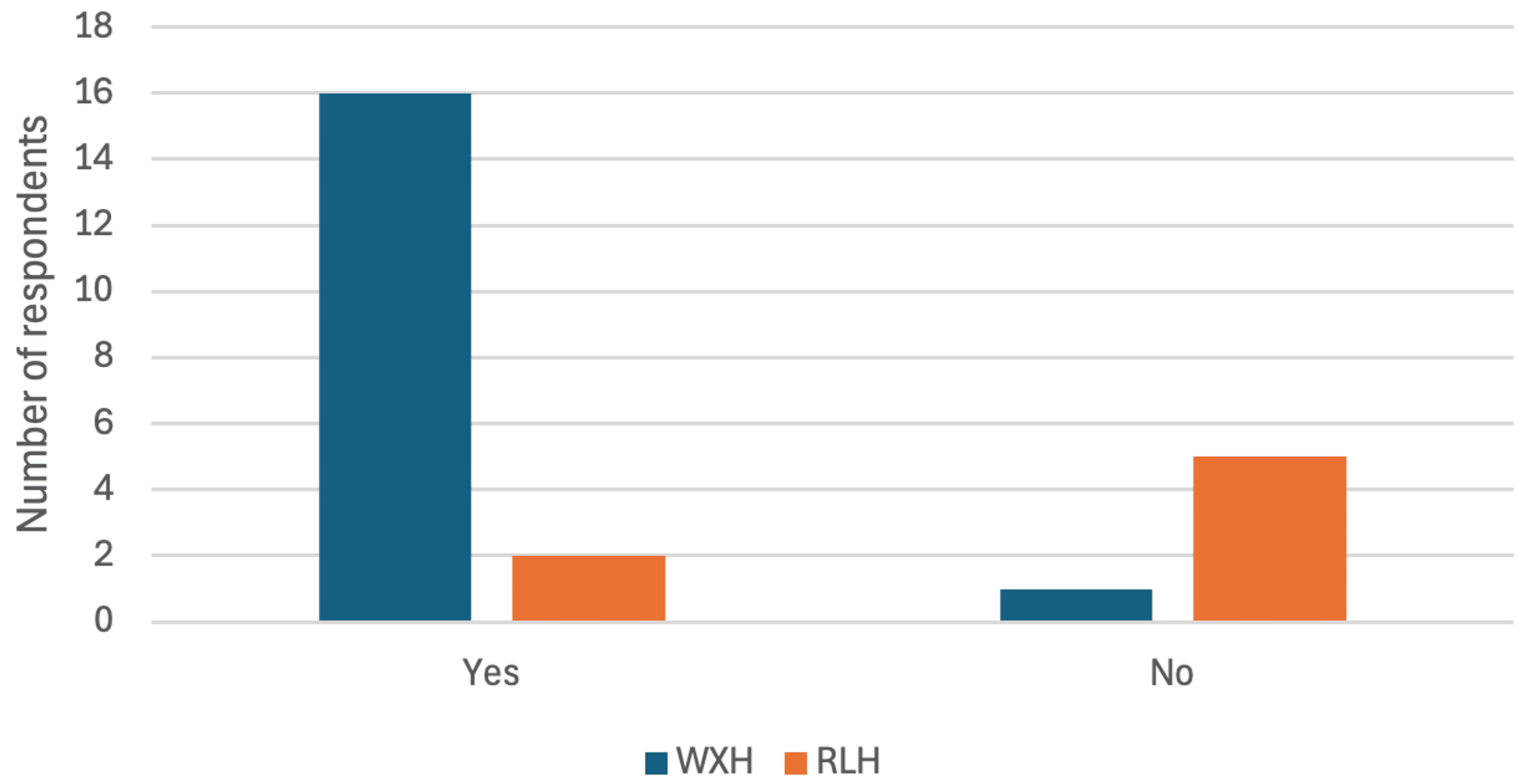
Survey Question: "Would you benefit from further training?" – Bar chart comparing responses between WXH and RLH radiographers. WXH: Whipps Cross Hospital; RLH: Royal London Hospital

Furthermore, 16 (94%) of the WXH respondents felt they would benefit from additional training. In contrast, only 2 (29%) of the RLH staff expressed a need for further training (Figure 4), indicating a better confidence level at RLH.

These findings emphasise the need for targeted training and improved support for radiographers at WXH to enhance the quality of radiographic imaging and reduce patient radiation risk.

A short-term review within the WXH department, assessing the diagnostic quality of intra-oral radiographs following the implementation of these interventions aimed to begin evaluating the effectiveness of the training programs, super user system, and rota sharing in improving the diagnostic quality of radiographs. These efforts continue to drive progress toward achieving consistent, high-quality radiography standards.

## Discussion

The results indicated a clear need for enhanced training and structured support for radiographers at WXH to achieve parity in intra-oral radiograph quality with RLH and meet the FGDP guidelines.

As WXH is a district general hospital with a smaller OMFS department, they take far less IOPAs than at RLH. Apart from this, the team at WXH consists of medical radiographers and has less exposure to dental radiography overall. This provides some insight into why radiographer confidence levels may be low. We identified that the main causes of the radiographs being nondiagnostic were due to either incorrect placement of the film or the positioning of the X-ray beam, which is a common cause found in other studies [15-16]. Carrying out training days at WXH allowed us to provide tailored teaching to the WXH radiography team, allowing us to develop their confidence, using equipment they would be using on a day-to-day basis.

The introduction of super users ensures that training is standardized and disseminated consistently across the team, especially as there is a rotation of junior staff. By sharing the OMFS rota, the allocation of a trained super user during clinic hours ensures that the quality of radiographs can be maintained without requiring clinician assistance.

These initiatives will not only improve the diagnostic quality of the radiographs but also boost the confidence of the radiographers, contributing to better patient outcomes.

Training improvements

To address the gaps identified, the liaison with the radiography department highlighted that previous training was carried out at RLH for the WXH team. They expressed that the equipment was different and that the team would benefit from training on site with the equipment that would be used during clinics.

A multi-disciplinary approach to the training was implemented which involved a tailored training day at WXH for the radiography staff. Theoretical knowledge was revised including understanding of the paralleling technique, patient positioning and highlighting the importance of reducing radiation exposure. This was followed by a practical session to allow the medical radiographers to become confident with the equipment available to them when taking IOPAs. The same dental chair, film holders, X-ray beam and rectangular collimator were used during the training session.

A decision between the radiography and OMFS team included the implementation of super users, who would be trained specifically for dental/oral and maxillofacial radiographs. The radiography team planned to allocate these ‘super users’ during busy OMFS clinics. These super users are now responsible for mentoring their peers, ensuring that knowledge is effectively transferred and applied in practice.

To ensure we meet the training needs of the radiography team, we implemented a plan for bi-annual radiography training to ensure super-users are up to date with training as well as any new members of the radiography team.

Rota implementation

The OMFS team at WXH already has a structured rota in place, outlining each clinic running within the week. Sharing of this rota with the radiography team ensures that super users are available during clinics, which has minimized disruptions in patient care by reducing the frequency of clinician intervention. This system also allows the medical radiographers to work more independently, knowing that trained support is available when necessary.

Limitations

One limitation of this study was the relatively small sample size. With only 100 radiographs being assessed across both sites, the data may not be completely representative. Also given that overall, fewer IOPAs are taken at WXH compared to RLH, we need to account that this, despite training, may be a leading cause for IOPAs not being diagnostically acceptable.

Also, the survey received a low response rate from RLH and may also not be representative of the whole radiography team there. As well as this survey data may be subject to self-reporting biases, as radiographers' perceptions of their own abilities and training may not always align with actual performance.

Finally, although a short-term review is showing promising findings as a result of our implementations, a longer-term, thorough second cycle is required to truly assess our interventions. Future cycles with larger sample sizes over longer periods of time will better assess the sustainability of our changes and provide an enhanced understanding of their effectiveness in improving radiograph quality.

## Conclusions

In conclusion, the analysis highlighted the need for improved training and support for medical radiographers tasked with taking intra-oral radiographs. These implementation of structured training, super users and rota sharing are anticipated to enhance both the diagnostic accuracy of radiographs and the confidence of the radiography team. A short-term review is already showing the positive outcomes of these interventions. Due to the small number of intra-oral radiographs taken at WXH, we can identify the need for a larger data set over a longer period of time to truly see the effect of the implementations. Future audit cycles will evaluate the long-term impact of these changes and explore additional areas for improvement.
